# Pain, sleep and emotional well-being explain the lack of agreement between physician- and patient-perceived remission in early rheumatoid arthritis

**DOI:** 10.1186/s41927-018-0024-9

**Published:** 2018-06-26

**Authors:** Samina A. Turk, Linda A. Rasch, Dirkjan van Schaardenburg, Willem F. Lems, Marjolein Sanberg, Lilian H. D. van Tuyl, Marieke M. ter Wee

**Affiliations:** 1 Department of Rheumatology, Amsterdam Rheumatology and immunology Center | Reade, PO box 58271, 1040 HG, Amsterdam, The Netherlands; 2 Department of Rheumatology, Amsterdam Rheumatology and immunology Center | VU University Medical Center, Amsterdam, Netherlands; 3 Department of Rheumatology, Amsterdam Rheumatology and immunology Center | Academic Medical Center, Amsterdam, Netherlands; 4 Department of Epidemiology and Biostatistics, VU University Medical Center, Amsterdam, Netherlands

**Keywords:** Rheumatoid arthritis, Disease-modifying antirheumatic drugs (Dmards), Patient reported outcomes, Fatigue, Physician agreement

## Abstract

**Background:**

Clinical response and remission are defined in multiple ways and measured with different instruments, resulting in substantial variation of the proportion of patients classified as being in remission. Therefore, the agreement between patient-perceived, physician-perceived remission and clinical response and remission definitions was determined in early rheumatoid arthritis (RA) patients. And secondly, differences in clinical and patient-reported outcomes, in patients in physician-perceived remission, between patients in and not in self-perceived remission were assessed.

**Methods:**

In 84 early RA patients, who received methotrexate and glucocorticoids, DAS44, ACR/EULAR Boolean-based remission, EULAR good and ACR70 response were determined after 12 weeks. Agreement between patient-perceived (phrased: “*Would you say that, at this moment, your disease activity is as good as gone?”*), physician-perceived remission (based on a visual analogue scale for global disease severity) and clinical response and remission definitions were calculated with the percentage of agreement and with kappa values (which corrects for change). In patients in physician-perceived remission, improvement in clinical and patient-reported outcomes (RAID) were compared between patients in and not in self-perceived remission.

**Results:**

Agreement between the assessed outcome measures differed enormously. The agreement between physician-perceived and patient-perceived remission was 64% (kappa 0.25, *p* < 0.01). Physician-perceived remission had the best agreement with EULAR good response (79%), and patient-perceived remission with EULAR good and ACR70 response (both 69%). Patients not in self-perceived remission improved less on RAID components, especially on pain, sleep and emotional well-being.

**Conclusion:**

One-third of the early RA patients disagreed with the physician on being in remission. Those patients had less improvement on RAID components, especially on pain, sleep and emotional well-being. Together with the variability in clinical response and remission definitions, these results highlight the need to increase patient involvement in their own health care decisions.

**Electronic supplementary material:**

The online version of this article (10.1186/s41927-018-0024-9) contains supplementary material, which is available to authorized users.

## Background

Since rheumatoid arthritis (RA) patients are at risk for joint damage due to inflammation [1], the treatment goal in these patients is to attain a state of absence of disease activity, or remission [2]. However, clinical response and remission are defined in multiple ways and measured with different instruments, resulting in substantial variation of the proportion of patients classified as being in remission [3, 4]. A particularly common difference is seen between the physician and the patients view on the RA disease activity [5–9].

The response to treatment as determined by the physician, is often based on the disease activity score (DAS), which is mainly based on physical examination and laboratory values [10, 11]. The DAS also contains a patient-reported outcome (PRO), i.e. the patient global assessment, however this global view lacks information on the patient’s perspective on remission [12]. Furthermore, PROs such as fatigue and physical well-being, which have a large impact on daily life, are not taken directly into account [7]. Nowadays, the importance of the patient’s perspective is increasingly recognized. Even though the patient’s perspective on remission is increasingly being studied and understood [12, 13], it is unknown which determinants of disease activity explain the lack of agreement between physician- and patient-perceived remission. Patient satisfaction, the relationship between patient and physician, and treatment compliance can all be improved when patient and physician agree on the state of the disease [14–17], which can be reached by taking the opinion of the patient into account and thus with applying shared decision-making [18–20].

The objective of this study was twofold. First, the frequencies were examined of patients that achieved physician-perceived remission, patient-perceived remission, DAS44 remission, European League Against Rheumatism (EULAR) good response, American College of Rheumatology (ACR) 70 response, and ACR/EULAR Boolean-based definition of remission [21–23]. With this data, the agreement between patient- and physician-perceived remission with and between the different clinical definitions of response and remission was determined. Second, the differences in clinical outcomes and PROs, in patients who did and did not agree with their physician on being in remission were assessed. Our hypothesis was that we would find significant differences in patients achieving remission according to the different response and remission criteria, compared to those who do not. Secondly, we hypothesised that there would be a lack of agreement between patient and physician perceived remission and several PROs.

## Methods

### Study population

The study population is part of a cohort of consecutive patients with early arthritis from the ‘Early Arthritis Cohort’ at Reade in Amsterdam, The Netherlands. This ongoing cohort includes patients aged 18 years and older with no prior treatment with disease-modifying antirheumatic drugs (DMARDs). Patients who fulfilled the ACR/ EULAR 2010 criteria for RA [24], and consented to start treatment with methotrexate (escalated to 25 mg/week) with 5 mg folic acid and glucocorticoids (30 mg/day tapered to 7,5 mg in 9 weeks) [25], between June 2014 and December 2016, were selected for inclusion. Approval was obtained from the local ethics committee (P0120, Ethics Committee of the Slotervaart Hospital and Reade, Amsterdam, The Netherlands) and all patients gave written informed consent according to the Declaration of Helsinki.

### Measurements

Patients were interviewed by research nurses, at baseline and after 12 weeks to record clinical characteristics as well as the DAS44. Erythrocyte sedimentation rate (ESR), C-reactive protein (CRP), rheumatoid factor (RF) and anti-citrullinated protein (ACPA) were determined. The Health Assessment Questionnaire (HAQ) and Rheumatoid Arthritis Impact of Disease (RAID) questionnaires were filled out [4, 26, 27]. The RAID evaluates the impact of RA on daily activities and comprises seven domains that are evaluated as continuous variables from 0 (best) to 10 (worst).

Patient- and physician perceived remission were determined after 12 weeks of treatment. To assess patient-perceived remission the following question was phrased: *“Would you say that, at this moment, your disease activity is as good as gone? Yes or no?”* [13]. Patients answering ‘yes’ were in ‘self-perceived remission’. Physician-perceived remission was assessed at the moment the physician assessed the patient in the outpatient clinic, using the ‘VAS physician’, phrased as: *“How active do you think the rheumatoid arthritis of your patient is today?”* and scored on a visual analogue (VAS) scale of 0–100 mm*.* Where a VAS ≤10 mm was defined as physician-perceived remission, according to the ACR/EULAR Boolean-based definition of remission [22].

Response after 12 weeks of treatment was determined, using the following clinical response and remission definitions: DAS44 remission (DAS44 < 1.6 points at week 12), EULAR good response (defined as DAS44 improvement of 1.2 points and a DAS44 score at week 12 ≤ 2.4) [23], ACR70 response [21], and ACR/EULAR Boolean-based remission [22].

### Statistical analyses

For descriptive purposes, mean (standard deviation (SD)), median [interquartile range (IQR)] or frequencies (percentages) were used. Differences between baseline and week 12 data were determined by the paired t-test when outcome variables were normally distributed. Otherwise, the Wilcoxon signed-rank test was applied.

First, the frequencies of patients who achieved DAS44 remission, EULAR good response, ACR70 response and Boolean-based remission were calculated, as well as the number of patients who were in physician- and patient-perceived remission. Second, the agreement of physician-perceived remission and patient-perceived remission with and between all clinical response and remission definitions were calculated, using the percentage of agreement as well as kappa values, according to the interpretation of Landis and Koch (<0 indicates no agreement, 0 to 0.2 slight, 0.21 to 0.40 fair, 0.41 to 0.60 moderate, 0.61 to 0.80 substantial and 0.81 to 1.0 as almost perfect agreement). Kappa can be interpreted as the percentage of agreement after correcting for chance [28, 29]. Third, analyses were performed in a subgroup of patients in physician-perceived remission. In this group, the differences between patients in and not in self-perceived remission, were assessed on several outcome measures: the improvement on clinical, laboratory and questionnaire data. This was analysed with the independent t-test (normal distribution) or the Mann-Whitney U test (skewed distribution). A *p*-value < 0.05 was considered statistically significant, and all analyses were performed with SPSS software (version 21).

## Results

### Total population

In total 84 patients with early RA of the ‘Early Arthritis Cohort’ were included. At baseline 10 patients did not complete the RAID questionnaire, and after 12 weeks three patients did not fill out the RAID questionnaire.

The mean (SD) age of the included patients was 50 (12) years, and 67% were female (Table 1). Mean (SD) DAS44 at baseline was 3.4 (1.2) and the seven questions on the RAID all had a median score between 4.0 and 7.0 at baseline. After 12 weeks of treatment, mean DAS44 (SD) improved to 1.4 (0.9) (*p* < 0.01), and all questions on the RAID improved to a median score between 2.0 and 4.0 (*p* < 0.01).

**Table 1:** Demographics and outcomes at baseline and after 12 weeks of treatment

	Baseline values			Values after 12 weeks of treatment		
	Total population *n* = 84	Patients in self-perceived remission at week 12, *n* = 45	Patients not in self-perceived remission at week 12, *n* = 39	Total population, *n* = 84	Patients in self-perceived remission at week 12, *n* = 45	Patients not in self-perceived remission at week 12, *n* = 39
Demographics						
Gender (female), *n* (%)	56 (66.7)	31 (69.9)	25 (64.1)			
Age (years)	50.0 (12.4)	50.4 (13.2)	49.4 (11.5)			
RF positive, *n* (%)	70 (83.3)	38 (84.4)	32 (82.1)			
ACPA positive, *n* (%)	72 (85.7)	39 (86.7)	33 (84.6)			
Symptom duration (months)	8.0 [3.5–20.0]	12.0 [4.0–22.0]	7.0 [3.0–18.0]			
Disease activity						
DAS44	3.4 (1.2)	3.2 (1.2)	3.6 (1.1)	1.4 (0.9)*	1.0 (0.6)	1.9 (0.9)‡
VAS global (mm)	62.0 [41.5–82.3]	50.0 [29.5–73.5]	70.0 [57.0–85.0]†	12.0 [5.0–42.8] *	5.0 [1.0–12.5]	40.0 [13.0–50.0]‡
TJC44ritchie	7.0 [3.3–10.8]	5.0 [3.0–10.0]	7.0 [5.0–11.0]	1.0 [0.0–2.0] *	0.0 [0.0–1.0]	2.0 [1.0–6.0]‡
SJC44ritchie	6.5 [3.0–13.0]	7.0 [2.5–13.0]	6.0 [3.0–12.0]	0.0 [0.0–2.0] *	0.0 [0.0–1.0]	2.0 [0.0–4.0]‡
ESR (mm/hour)	20.0 [9.0–32.8]	15.0 [7.0–30.0]	23.0 [15.0–40.0]†	7.0 [5.0–12.0] *	7.0 [3.5–12.0]	8.0 [5.0–12.0]
CRP (mg/l)	7.7 [3.9–25.8]	5.5 [3.5–24.0]	8.3 [4.3–33.0]	2.0 [1.1–3.6] *	1.8 [1.1–3.9]	2.0 [0.9–3.6]
Patient Reported Outcomes Measures						
RAID pain (0–10)	7.0 [5.8–8.0]	7.0 [4.0–8.0]	7.5 [6.0–8.3]	2.0 [1.0–4.5] *	1.0 [0.0–2.0]	4.0 [2.5–6.0]‡
RAID FDA (0–10)	6.0 [4.0–8.0]	5.5 [2.0–8.0]	7.0 [5.0–8.3]†	2.0 [0.0–4.5] *	1.0 [0.0–2.0]	5.0 [3.0–7.5]‡
RAID fatigue (0–10)	6.5 [3.0–8.0]	5.5 [2.0–8.0]	7.0 [4.8–9.0]†	4.0 [2.0–7.0] *	2.0 [0.0–5.8]	5.0 [3.0–7.5]‡
RAID sleep (0–10)	7.0 [2.0–8.0]	7.0 [2.0–8.0]	6.5 [3.8–8.0]	2.0 [0.0–6.0] *	1.0 [0.0–4.0]	5.0 [2.0–7.0]‡
RAID physical well-being (0–10)	4.0 [2.0–7.0]	3.0 [1.3–6.0]	5.0 [3.0–7.0]†	3.0 [1.0–5.0] *	1.0 [0.0–3.0]	4.0 [2.0–5.0]‡
RAID emotional well-being (0–10)	5.0 [2.0–7.0]	5.0 [1.3–6.8]	6.0 [3.0–8.0]	2.0 [0.0–5.0] *	1.0 [0.0–3.0]	4.0 [2.0–6.0]‡
RAID coping (0–10)	5.0 [2.0–7.0]	3.0 [1.3–7.0]	5.0 [3.0–7.3]	2.0 [0.0–4.0] *	0.5 [0.0–2.0]	3.0 [2.0–5.0]‡
HAQ (0–3)	0.9 [0.5–1.5]	0.8 [0.3–1.3]	1.3 [0.6–0.8]†	0.2 [0.0–0.6] *	0.0 [0.0–0.3]	0.5 [0.1–0.9]‡

Numbers are presented as mean (SD) or median [IQR] unless otherwise stated

*ACPA* anti-citrullinated protein, *CRP* C-reactive protein, *DAS44* disease activity score of 44 joints, *ESR* erythrocyte sedimentation rate, *FDA* functional disability assessment, *HAQ* Health Assessment Questionnaire, *IQR* interquartile range, *l* liter, *mg* milligram, *mm* millimeter, *n* number, *RAID* Rheumatoid Arthritis Impact of Disease questionnaire, *RF* rheumatoid factor, *SD* standard deviation, *SJC44* swollen joint count of 44 joints, *TJC44* tender joint count of 44 joints, *VAS* visual analogue scale

*Significant improvement (*p* < 0.05) for the total population, between baseline and 12 weeks after treatment

† Significant difference (*p* < 0.05) in baseline values between patient in and patient not in self-perceived remission after 12 weeks of treatment

‡ Significant difference (*p* < 0.05) in week 12 values between patients in and patient not in self-perceived remission after 12 weeks of treatment

### Patients who fulfil the different response and remission criteria

After 12 weeks of treatment, 65 patients (77%) reached an EULAR good response, 25 patients (30%) an ACR70 response and 23 patients (27%) were in Boolean-based remission (Additional file 1). Fifty-one patients (61%) reached DAS44 < 1.6 and 50 patients (60%) had a HAQ score < 0.5.

All analyses were repeated for a cut-off of VAS physician remission of ≤20 mm and showed similar results (data not shown) .

### Remission according to the physician and patient

According to physician-perceived remission, 55 patients (66%) were in remission after 12 weeks of treatment.

Patients in self-perceived remission versus those not at week 12 (*n* = 45, 54%) had a significantly lower DAS, tender joint count (TJC) and swollen joint count (SJC) of 44 joints and scored lower on all questions on the RAID (Table 1). The VAS physician was lower in patients who perceived themselves in remission, compared to those who did not 5.0 [2.5–9.5] versus 13.0 [7.0–34.0] (*p* < 0.01), respectively. Differences at baseline were seen between patients in and not in self-perceived remission after 12 weeks. Patients in self-perceived remission scored significantly lower at baseline on the VAS global, the HAQ, and on the RAID questions about functional disability assessment, fatigue and physical well-being. Of the clinical outcomes, only ESR was significantly lower in patients in self-perceived remission compared to those who were not: 15.0 [7.0–30.0] versus 23.0 [15.0–40.0] mm/hour (*p* < 0.01; Table 1), respectively.

### Agreement between physician, patient and clinical response and remission definitions

The agreement between physician-perceived remission and patient-perceived remission was 67% (kappa 0.32, *p* < 0.01; Table 2).

**Table 2:** Agreement between different definitions of response and remission

	Physician-perceived remission	Patient-perceived remission	DAS44 remission	EULAR good response	ACR70 response	Boolean remission
Physician- perceived remission	x	67%	74%	79%	60%	57%
		Ƙ = 0.318	Ƙ = 0.439	Ƙ = 0.484	Ƙ = 0.281	Ƙ = 0.248
		*P* = 0.003	*P* < 0.001	*P* < 0.001	*P* = 0.001	*P* = 0.002
Patient-perceived remission	67%	x	46%	69%	69%	67%
	Ƙ = 0.318		Ƙ = 0.516	Ƙ = 0.356	Ƙ = 0.398	Ƙ = 0.354
	*P* = 0.003		*P* < 0.001	*P* < 0.001	*P* < 0.001	*P* < 0.001
DAS44 remission	74%	46%	x	83%	64%	67%
	Ƙ = 0.439	Ƙ = 0.516		Ƙ = 0.622	Ƙ = 0.343	Ƙ = 0.392
	*P* < 0.001	*P* < 0.001		*P* < 0.001	*P* < 0.001	*P* < 0.001
EULAR good response	79%	69%	83%	x	52%	50%
	Ƙ = 0.484	Ƙ = 0.356	Ƙ = 0.622		Ƙ = 0.220	Ƙ = 0.199
	*P* < 0.001	*P* < 0.001	*P* < 0.001		*P* = 0.001	*P* = 0.002
ACR70 response	60%	69%	64%	52%	x	74%
	Ƙ = 0.281	Ƙ = 0.398	Ƙ = 0.343	Ƙ = 0.220		Ƙ = 0.359
	*P* = 0.001	*P* < 0.001	*P* < 0.001	*P* = 0.001		*P* = 0.001
Boolean remission	57%	67%	67%	50%	74%	x
	Ƙ = 0.248	Ƙ = 0.354	Ƙ = 0.392	Ƙ = 0.199	Ƙ = 0.359	
	*P* = 0.002	*P* < 0.001	*P* < 0.001	*P* = 0.002	*P* = 0.001	

Numbers are presented as level of agreement (%), kappa value (K) and *p*-value (P)

Physician-perceived remission was defined as a VAS of ≤10 mm as answer to the question: “*How active do you think the rheumatoid arthritis of your patient is today?”*

Patient-perceived remission was defined as “*yes”* or “*no”* as answer to the question*: “Would you say that, at this moment, your disease activity is as good as gone?”*

*ACR70* American College of Rheumatology 70 response, *DAS44* disease activity score of 44 joints, *EULAR* European League Against Rheumatism, *mm* millimeter

The physician-perceived remission had the best agreement with EULAR good response: 79% agreement, with a kappa of 0.48; *p* < 0.01 (Table 2).

The agreement with patient-perceived remission was highest for EULAR good response as well as ACR70 response: both 69% (kappa 0.36 (*p* < 0.01) and 0.40 (*p* < 0.01), respectively). The agreement with Boolean-based remission was slightly lower and the lowest agreement was seen with DAS44 remission.

Concordance between the different clinical response and remission definitions differed enormously. For example, the agreement between EULAR good response and DAS44 remission was 83% (kappa 0.62, *p* < 0.01), but the agreement between EULAR good response and ACR70 response was 52% (kappa 0.22, *p* < 0.01; Table 2). Agreement between physician- and patient perceived remission differed as well within the different response and remission criteria (Additional file 1).

### Discordance between physicians and patients in remission

**Fig. 1:**
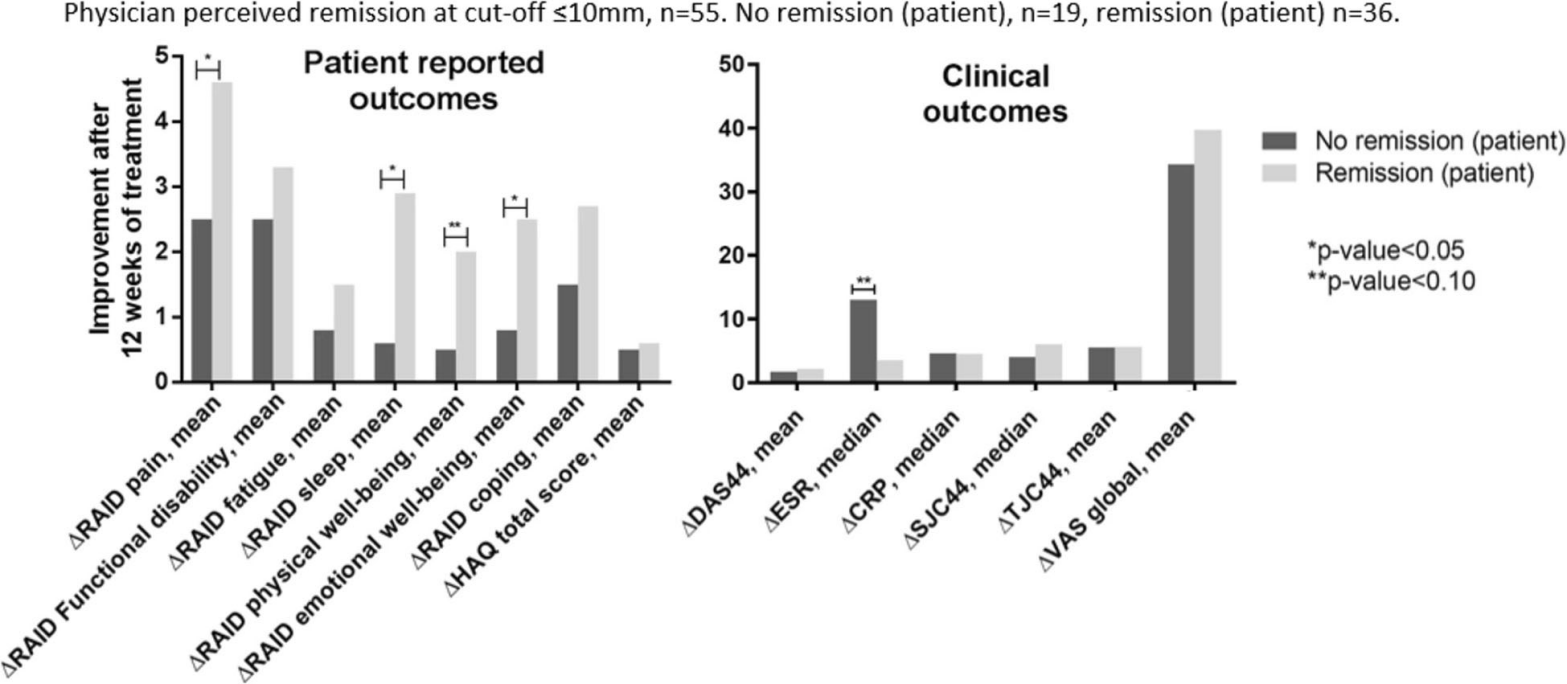
Comparison of improvement in patient-reported and clinical outcomes after 12 weeks of treatment, in patients in physician-perceived remission, who were in and not in patient-perceived remission

In this subgroup analyses, only patients in physician-perceived remission were included. Patients in self-perceived remission showed the same improvement in DAS44, tender and swollen joint count after 12 weeks of treatment, compared to those who categorized themselves not in self-perceived remission (Fig. 1). A trend was seen in the difference on the change in ESR: 3.5 versus 13.0 mm/hour (*p* = 0.07). An improvement on all questions on the RAID was seen, however, patients in self-perceived remission improved significantly more on the question about sleep, compared to patients not in self-perceived remission: 2.9 versus 0.6 (*p* = 0.01). A significant difference was seen in improvement on the questions pain and emotional well-being, between patients in self-perceived remission compared to those not in self-perceived remission (*p*-value for both 0.04). Looking retrospectively at the baseline values for the differences between patients in and not in self-perceived remission no significant differences were found (Table 3) [30].

**Table 3:** Differences in baseline values of patients in physician-perceived remission, stratified into patients in and not in self-perceived remission after 12 weeks of treatment

	In physician-perceived remission, *n* = 55	
	In patient-perceived remission, *n* = 36	Not in patient-perceived remission, *n* = 19
DAS44	3.0 (1.1)	3.2 (1.0)
VAS global (mm)	47.5 [28.3–72.0]	66.0 [50.0–76.0]
TJC44ritchie	4.0 [3.0–9.0]	6.0 [4.0–11.0]
SJC44ritchie	6.0 [2.3–12.0]	6.0 [3.0–12.0]
ESR (mm/hour)	14.5 [8.0–31.0]	20.0 [12.0–40.0]
CRP (mg/l)	10.7 [4.2–24.8]	7.2 [3.9–32.0]
RAID pain	7.0 [3.0–8.0]	7.0 [4.8–8.0]
RAID FDA	6.0 [2.0–7.0]	5.0 [4.0–8.0]
RAID fatigue	5.0 [1.0–8.0]	6.0 [3.0–8.0]
RAID sleep	6.0 [2.0–8.0]	5.5 [2.0–7.3]
RAID physical well-being	3.0 [1.0–6.0]	3.5 [3.0–6.8]
RAID emotional well-being	3.0 [1.0–7.0]	4.0 [2.5–7.0]
RAID coping	3.0 [1.0–8.0]	5.0 [2.5–8.0]
HAQ	0.6 [0.1–1.1]	0.8 [0.4–1.5]

Numbers are presented as mean (SD) or median [IQR] where appropriate

*CRP* C-reactive protein, *DAS44* disease activity score of 44 joints, *ESR* erythrocyte sedimentation rate, *FDA* functional disability assessment, *HAQ* Health Assessment Questionnaire, *IQR* interquartile range, *l* liter, *mg* milligram, *mm* millimeter, *n* number, *RAID* Rheumatoid Arthritis Impact of Disease questionnaire, *SD* standard deviation, *SJC44* swollen joint count of 44 joints, *TJC44* tender joint count of 44 joints, *VAS* visual analogue scale

*Significant difference (*p* < 0.05) in baseline values for patients in and not in self-perceived remission after 12 weeks of treatment

## Discussion

More than one-third of early RA patients disagreed with their physician on being in remission after 12 weeks of treatment. The agreement between physician and patient was higher in patients who did achieve DAS44 remission, ACR70 response or were in Boolean-based remission. Patients who judged themselves as not being in self-perceived remission showed less improvement on the RAID questions on sleep, pain and emotional well-being, compared with patients who judged themselves as being in self-perceived remission.

In this study all patients received the same anti-rheumatic treatment, which led to an improvement in disease activity of all patients. The improvement of mean two points in the DAS44 score after 12 weeks of treatment was similar to the results of the COBRA-light trial. The improvement on RAID was in agreement with the results of the study of Ledingham et al. [31, 32]. Clinical response and remission definitions in RA are defined in several ways and the stringency of these different definitions has been shown to vary widely and lead to enormous differences in results, which is comparable to our results as 61% reached DAS44 remission, while 27% of the patients achieved Boolean-based remission [33].

Our results showed a similar percentage of agreement between physician- and patient-perceived remission, as in existing literature an agreement between 51 and 79% is seen [5–9]. For example, the Danish DANBIO registry found a 51% agreement between 8300 RA patients and physicians. Disagreement in this study was defined as a difference of >20 mm on the global assessment between the patient and the physician [8]. However, the DANBIO registry described patients with a mean disease duration of 7 years and patients with lower disease activity, while the current study included patients at the onset of RA, who generally have a higher disease activity. The higher agreement between patients and physicians in the present study was probably due to higher disease activity scores, as a higher swollen joint count is found to be associated with lower odds of discordance [5]. This is also visible in the variability of agreement between physicians and patients within different response and remission definitions, as agreement was higher in patients who achieved ACR70 response or Boolean remission. In the present study, both patients and physicians had the best agreement with EULAR good response, which was predictable as (improvement in) DAS is the most commonly used measurement in clinical practice [33].

A difference was not found in the improvement of DAS44 score, SJC or TJC between patients who did and did not agree with the physician on being in remission. But, where physicians focus on disease activity (inflammation), patients also incorporate other domains [7, 10, 11]. Patients who did not agree with their physician on being in remission did show less improvement on components of the RAID about sleep, pain and emotional well-being. A non-significant difference in fatigue was found between patients who did and did not perceive themselves in remission. This is in contrast with other studies, in which fatigue was an important explanation of patients perception of disease activity [7, 34]. However, in these studies fatigue covered fatigue and sleep problems, which was separated in our study. This might explain the difference, however instead of the disease itself, the side effects of medication, especially glucocorticoids, can also explain a part of the sleep difficulties and fatigue symptoms. In this study, all patients received the same dose of glucocorticoids, however some patients may experience more side effects than others. Patients who did not agree also showed more improvement in ESR after 12 weeks. These patients showed a trend of a higher ESR at baseline, but no significant difference was found after 12 weeks of anti-rheumatic treatment. We hypothesized that patients who did not perceive themselves in remission, had more low grade inflammation during the 12 weeks, which might be associated with more fatigue and sleep difficulties [34, 35]. At baseline their mean ESR was higher, but they improved more in ESR to reach the same ESR levels at week 12 as patients who were in self-perceived remission. However, this was not seen for CRP levels. The comparison between the RAID score and the discordance of physician- and patient-perceived remission has not yet been performed before, as far as we know, which is a strength of this study. Our study has some limitations. First, there is no widely accepted cut-off point for discordance and therefore we used the same cut-off as the ACR/EULAR Boolean-based definition of remission [23] were a VAS ≤ 10 mm was accepted to define physician-perceived remission. We also performed a sensitivity analysis with a cut-off VAS ≤ 20 mm, which showed similar results. However, similar results were found in a study performed in 800 RA patients, where a median VAS physician of 15 mm was found in patients who were in physician-perceived remission [9]. Second, the number of patients included in this study was small, which influences the possibility to find significant relationships in the data. However, this was of minimal influence as similar results in previous articles were found [5–9]. Finally, we did not take adverse effects as well as other concomitant diseases into account that could influence the self-assessment of RA activity. However, the measurements of RA disease activity that are used in general care, do not consider this aspect either [10]. Nonetheless, for future perspectives questions on adverse effects, comorbidity and mental state might be useful. Future studies are needed to confirm our findings and to determine the optimal set of patient-reported outcomes. And eventually to compare the current treat-to-target treatment strategy with patient-reported outcome guided treatment.

## Conclusions

In conclusion, more than one-third of the patients disagreed with their physician on being in remission. This might have consequences for patient satisfaction, the relationship between patient and physician and treatment compliance of the patient. Patients who disagreed with their physician on being in remission showed less improvement on questions about sleep, pain and emotional well-being of the RAID. However, not only patients and physicians showed discordance, there were also many differences between clinical response and remission definitions. This makes it necessary to increase patient involvement in their own health care decisions, improving shared decision making.

## Additional file

Additional file 1: Agreement between physician- and patient-perceived remission in different clinical definitions of response and remission. (PDF 164 kb)

## Abbreviations

ACPA: Anti-citrullinated protein
ACR: American College of Rheumatology
CRP: C-reactive protein
DAS: Disease activity score
DMARDs: Disease modifying antirheumatic drugs
ESR: Erythrocyte sedimentation rate
EULAR: European League Against Rheumatism
HAQ: Health Assessment Questionnaire
IQR: Interquartile range
PRO: Patient-reported outcome
RA: Rheumatoid arthritis
RAID: Rheumatoid Arthritis Impact of Disease
RF: Rheumatoid factor
SD: Standard deviation
SJC: Swollen joint count
TJC: Tender joint count
VAS: Visual analogue scale
