# Supplementary material for: Pain, sleep and emotional well-being explain the lack of agreement between physician- and patient-perceived remission in early rheumatoid arthritis
Source: BMC Rheumatol. 2018 Jun 26;2:16. doi: 10.1186/s41927-018-0024-9 (PMC6390551; doi:10.1186/s41927-018-0024-9)
Supplement: Supplementary file 1 — Agreement between physician- and patient-perceived remission in different clinical definitions of response and remission. (PDF 164 kb) [file 41927_2018_24_MOESM1_ESM.pdf]

Additional file 1. Agreement between physician- (VAS ≤10 mm) and patient-perceived remission in different clinical definitions of response and remission.

Overall agreement between physician- and patient-perceived remission: 67%
Agreement between physician- and patient-perceived remission in patients in Boolean remission: 78%
Agreement between physician- and patient-perceived remission in patients with a good EULAR response: 65%
Agreement between physician- and patient-perceived remission in patients with an ACR70 response: 80%

Physician remission	Remission according to physician (n=84)		
	Remission physician	No remission physician	Total
Patient remission, n(%)	36 (66)	9 (31)	45 (54)
No patient remission, n(%)	19 (35)	20 (69)	39 (46)
Total, n(%)	55 (65)	29 (36)	84

Boolean remission	Remission (n=23, 27%)			No remission (n=61, 73%)		
	Remission physician	No remission physician	Total	Remission physician	No remission physician	Total
Patient remission, n(%)	18 (86)	2 (100)	20 (87)	18 (53)	7 (26)	25 (41)
No patient remission, n(%)	3 (14)	0 (0)	3 (13)	16 (47)	20 (74)	36 (59)
Total, n(%)	21 (91)	2 (9)	23	34 (56)	27 (44)	61

EULAR response	Good (n=65, 77%)			Moderate (n=12, 14%)			None responder (n=7, 8%)		
	Remission physician	No remission physician	Total	Remission physician	No remission physician	Total	Remission physician	No remission physician	Total
Patient remission, n(%)	35 (69)	7 (50)	42 (65)	1 (33)	1 (11)	2 (17)	0 (0)	1 (17)	1 (14)
No patient remission, n(%)	16 (31)	7 (50)	23 (35)	2 (67)	8 (89)	10 (83)	1 (100)	5 (83)	6 (86)
Total, n(%)	51 (78)	14 (22)	65	3 (25)	9 (75)	12	1 (14)	6 (86)	7

ACR response	ACR70 (n=25, 30%)			ACR50, but not ACR70 (n=10, 12%)			ACR20, but not ACR50 (n=20, 24%)			ACR non-responder (n=29, 35%)		
	Remission physician	No remission physician	Total	Remission physician	No remission physician	Total	Remission physician	No remission physician	Total	Remission physician	No remission physician	Total
Patient remission, n(%)	20 (87)	2 (100)	22 (88)	3 (50)	3 (75)	6 (60)	3 (25)	1 (13)	4 (20)	10 (71)	3 (20)	13 (45)
No patient remission, n(%)	3 (13)	0 (0)	3 (12)	3 (50)	1 (25)	4 (40)	9 (75)	7 (88)	16 (80)	4 (29)	12 (80)	16 (55)
Total, n(%)	23 (92)	2 (8)	25	6 (60)	4 (40)	10	12 (60)	8 (40)	20	14 (48)	15 (52)	29
